# Recommendations for microbiological sampling in infected hard-to-heal wounds: an Italian association for cutaneous ulcers (AIUC) perspective

**DOI:** 10.3389/fsurg.2026.1908843

**Published:** 2026-09-30

**Authors:** Sara Carella, Barbara De Angelis, Barbara Nardi, Francesco Branda, Diego Ceci, Caterina Borghetti, Alessandra Mercuri, Marino Cordellini, Cosimo Maglio, Monica Gonnelli, Riccardo Garcea, Giancarlo Ceccarelli

**Affiliations:** 1 Department of Surgery, Complex Unit of Plastic and Reconstructive Surgery, USL Umbria 1, Perugia, Italy; 2 Complex Wounds Unit, Policlinico Casilino, University of Tor Vergata, Rome, Italy; 3 Microbiology Laboratory, Assisi Hospital, USL Umbria 1, Assisi, Assisi, Perugia, Italy; 4 Unit of Medical Statistics and Molecular Epidemiology, University Campus Bio-Medico, Rome, Italy; 5 Department of Medical and Surgical Sciences and Translational Medicine, Faculty of Medicine and Psychology, Sapienza University of Rome, Sant'Andrea Hospital, Rome, Italy; 6 Department of Medicine, Departmental Simple Structure (S.S.D.) of Angiology, San Matteo degli Infermi Hospital, USL Umbria 2, Spoleto, Italy; 7 Department of Medicine and Surgery, Complex Unit of Infectious Diseases, University Hospital of Perugia, Perugia, Italy; 8 Complex Wounds and Diabetic Foot Clinic, Emergency General Surgery Unit, Ospedali Riuniti della Valdichiana Senese, Montepulciano, Siena, Azienda USL Toscana Sud Est, Italy; 9 Wound Care and Diabetic Foot Clinic and Nursing Clinic for Complex Skin Lesions, Ospedali Riuniti della Valdichiana Senese, Montepulciano, Siena, Azienda USL Toscana Sud Est, Italy; 10 Infirmary of the General Command of the Carabinieri, Arma dei Carabinieri, Rome, Italy; 11 Department of Public Health and Infectious Diseases, Sapienza University of Rome, Azienda Policlinico Umberto I, Rome, Italy

**Keywords:** infected hard-to-heal wounds, laboratory-methods, biopsy, swab, recommendations, artificial intelligence, medico-legal implications

## Abstract

Chronic and hard-to-heal wounds represent a significant and growing healthcare burden, frequently complicated by infection and impaired healing. Despite the availability of international guidelines, microbiological assessment in clinical practice remains highly heterogeneous, with substantial variability in sampling techniques, timing, and interpretation of results. This variability occurs within a broader One Health context, where antimicrobial resistance and microbial ecology are shaped by interconnected human, environmental, and healthcare factors. The present recommendations, developed by the Italian Association for Cutaneous Ulcers (AIUC), aim to provide a structured and operational framework for microbiological sampling in infected hard-to-heal wounds. A multidisciplinary expert panel conducted a structured review of the literature and developed consensus-based recommendations through iterative discussion. The resulting framework addresses key aspects of the diagnostic pathway, including indications for sampling, selection of techniques (swab, tissue biopsy, and fine needle aspiration), standard operating procedures, and principles for the interpretation of microbiological findings. The recommendations emphasise a context-driven approach to sampling, recognising the complementary roles of different techniques according to clinical presentation and depth of infection. Particular attention is given to the distinction between colonisation and infection, the limitations of quantitative thresholds, and the impact of biofilm, polymicrobial interactions, and host-related factors on diagnostic accuracy and antimicrobial response. The document also highlights the importance of integrating microbiological data with clinical assessment to support appropriate antimicrobial stewardship. By translating existing evidence into practical and reproducible procedures, these recommendations aim to reduce variability in clinical practice and improve the quality of microbiological diagnostics in wound care. Within a One Health perspective, standardised and reliable microbiological data may also support surveillance of antimicrobial resistance and inform more integrated infection control strategies. The proposed framework supports more consistent clinical decision-making and may contribute to optimising antimicrobial use and patient outcomes across diverse healthcare settings.

## 1 Introduction

Chronic wounds represent a relevant and still largely underestimated public health issue, associated with significant clinical, functional, and economic implications (1, 2). Despite the adoption of multidisciplinary care pathways and the availability of multiple therapeutic approaches, in high-income countries approximately 1%–2% of the population is estimated to develop, during their lifetime, slow- or non-healing cutaneous lesions, frequently characterized by a complex and recurrent course (1, 3). The overall disease burden is further increased by the presence of comorbidities such as diabetes mellitus, peripheral arterial disease, chronic venous insufficiency, neuropathies, and age-related frailty (4, 5).

Wound healing is a phase-dependent and multifactorial process—comprising hemostasis, inflammation, proliferation, and remodelling—in which the local microbial ecology represents a primary and dynamic biological determinant. The skin, including ulcerated surfaces, harbors a microbiota characterized by high taxonomic and functional complexity, with trophic and communicative networks that modulate inflammation, angiogenesis, and re-epithelialization. Systemic or topical antimicrobial interventions, particularly when non-selective, may induce dysbiosis, suppress mutualistic microbial populations, promote the selection of a clinically relevant resistome, and ultimately negatively impact wound healing outcomes (6, 7).

Biofilm has been reported in approximately 78.2% of chronic wounds, although prevalence estimates vary among studies (8). These structured microbial communities, embedded within an extracellular polymeric substance matrix, generate microenvironments characterized by gradients of nutrients and oxygen, thereby conferring tolerance to antimicrobial agents and protection from host immune responses. Within this ecological context, interspecific interactions—including competition, cooperation, and quorum sensing interference—play a key role in microbial persistence and pathogenicity, and may also represent potential therapeutic targets (8, 9).

Within this complex biological and clinical framework, the accurate identification and management of infection represent a critical determinant of patient outcomes. However, microbiological assessment of hard-to-heal wounds remains highly heterogeneous in clinical practice. Variability in sampling techniques, timing of collection, and laboratory processing leads to inconsistent diagnostic accuracy, difficulty in distinguishing colonisation from infection, and inappropriate antimicrobial use. In addition, pre-analytical factors and differences in interpretative criteria further contribute to variability and limit the reproducibility of microbiological findings (10–12).

Despite the availability of international guidelines and position statements, there is currently no universally standardised and operational framework for microbiological sampling in chronic wounds. Existing approaches are often operator-dependent and not consistently applicable across different healthcare settings, including hospital, outpatient, and community care. This lack of standardisation represents a significant gap in clinical practice, with implications for antimicrobial stewardship, clinical outcomes, and medico-legal risk.

To address this unmet need, the present document reports expert-panel recommendations developed by the Italian Association for Cutaneous Ulcers (AIUC), with the aim of standardising microbiological sampling procedures in infected hard-to-heal wounds and providing a structured and reproducible clinical framework applicable across different healthcare settings.

## 2 Materials and methods

A multidisciplinary expert panel, comprising all manuscript authors, was convened to develop recommendations for microbiological sampling in infected hard-to-heal wounds. The panel included specialists in plastic and reconstructive surgery, general surgery, vascular surgery, infectious diseases, clinical microbiology, epidemiology, wound care nursing, and medico-legal medicine, ensuring a comprehensive and multidisciplinary perspective. The panel operated within the AIUC network, involving representatives from different regional healthcare settings.

A structured desk review of the literature was conducted, focusing on international guidelines, position statements, and relevant scientific evidence, including documents from EWMA, IWII, IDSA, and other authoritative sources. The objective of this review was to identify current standards, evaluate methodological variability, and highlight critical gaps in microbiological sampling practices.

Panel members met online approximately every two weeks throughout the manuscript development process. During these sessions, panel members analysed key methodological aspects of microbiological sampling, including sampling techniques (swab, tissue biopsy, fine needle aspiration), timing in relation to debridement and antimicrobial therapy, pre-analytical variables, laboratory processing, and interpretation of microbiological findings. Draft recommendations were discussed, revised, and refined iteratively to ensure consistency with the available evidence and their applicability in clinical practice. Successive manuscript drafts incorporated the agreed revisions until unanimous agreement was reached among all panel members. Particular attention was given to the standardisation of procedures, clinical applicability across different healthcare settings (hospital, outpatient, and community care), and integration with antimicrobial stewardship principles. The final version of the manuscript was reviewed and approved by all authors before submission.

The final framework was structured into operational sections, including: (i) sampling techniques and indications, (ii) standard operating procedures, (iii) interpretation of microbiological findings, and (iv) integration with clinical and microbiological decision-making processes.

In addition, the framework incorporates emerging technological perspectives, including artificial intelligence–based tools for wound assessment and monitoring, and considers medico-legal implications related to diagnostic standardisation and procedural traceability.

The primary objectives of the consensus process were:

(1) to reduce variability in microbiological sampling practices;
(2) to improve diagnostic appropriateness and clinical interpretation;
(3) to support antimicrobial stewardship;
(4) to provide a structured and reproducible operational reference across different healthcare settings.

### 2.1 Limitations

The present recommendations were developed through structured multidisciplinary expert discussions rather than a formal consensus methodology, such as a Delphi process or nominal group technique. The recommendations were informed by a structured review of the available scientific literature, relevant international guidelines, and proposed standard operating procedures (SOPs); however, the literature search was not designed as a fully systematic review, and no formal evidence-grading framework (e.g., GRADE) was applied to assess the quality of evidence or the strength of individual recommendations. Consequently, these recommendations should be regarded as expert-panel recommendations aimed at supporting clinical decision-making and promoting the standardization of microbiological sampling in infected hard-to-heal wounds. Future updates of this document may incorporate formal consensus methodologies and evidence-grading systems as additional evidence becomes available.

## 3 Expert-panel recommendations for microbiological sampling

Based on the available evidence and the expert-panel process described above, the AIUC working group developed a set of structured recommendations for microbiological sampling in infected hard-to-heal wounds.

These recommendations are intended to provide a practical and operational framework for clinical practice, addressing key methodological aspects including sampling techniques, indications, timing, pre-analytical procedures, and interpretation of microbiological findings.

The recommendations are organised into thematic sections reflecting the diagnostic pathway, from sample collection to clinical interpretation, with the aim of ensuring consistency, reproducibility, and applicability across different healthcare settings.

Where appropriate, specific procedural steps are presented as standard operating procedures (SOPs) to facilitate implementation in routine clinical practice.

The SOPs presented in this document are intended to provide a standardized operational description of established microbiological sampling procedures. They were compiled by integrating procedural recommendations from the available scientific literature, international guidance, and routine microbiological practice, with the aim of facilitating their consistent implementation in routine clinical care.

For ease of implementation, the principal expert-panel recommendations are summarised below.

**Key Expert-Panel Recommendations**

1. Microbiological sampling is recommended when clinical signs of infection are present.
2. Tissue biopsy should be considered the gold standard for microbiological diagnosis of deep wound infections whenever clinically feasible.
3. Swab sampling remains an appropriate alternative when tissue biopsy is not feasible.
4. Fine-needle aspiration may be considered for selected deep or localised infections.
5. Standardised specimen collection, pre-analytical handling, transport, and laboratory processing are essential to ensure reliable microbiological results.
6. Microbiological findings should always be interpreted in conjunction with the clinical presentation.
7. Standardised microbiological sampling supports antimicrobial stewardship and epidemiological surveillance.

The following sections detail these recommendations, structured according to the diagnostic pathway and encompassing sampling techniques, procedural steps, and the clinical interpretation of microbiological findings.

### 3.1 Sampling collection

#### 3.1.1 Swab

##### 3.1.1.1 General considerations

Swab sampling involves the collection of biological material for microbiological purposes. It is considered a semi-quantitative method, as it does not allow for reliable bacterial quantification and may yield positive results due to the presence of superficial contaminants, particularly aerobic Gram-positive cocci (e.g., Staphylococcus spp. and Streptococcus spp) (13–16). Although swab sampling shows lower sensitivity and specificity compared with tissue biopsy, it offers the advantage of ease of performance, making it suitable for routine use in outpatient settings (17). The most commonly used swabs are made of cotton or alginate; the collected material is subsequently released into an appropriate transport medium, allowing dispersion of the sampled microbial load for laboratory analysis (13, 18, 19).

##### 3.1.1.2 Indications for swab sampling (17)

When should a wound swab be performed?

Main indications include:

- Clinical signs and symptoms of infection, such as delayed wound healing, presence of pus or abscess, erythema, induration, cellulitis, wound deterioration or enlargement, increased volume of exudate, malodour, increased or altered pain, pocketing, friable granulation tissue with a tendency to bleed, and necrosis;
- Clinical conditions, such as reduced or impaired immune response;
- Epidemiological reasons, including suspicion of colonisation or infection by multidrug-resistant organisms (e.g., MRSA, ESBL-producing bacteria).

##### 3.1.1.3 Sampling techniques

The two main validated wound swab sampling techniques are (Table 1):

**Table 1:** Wound swab sampling techniques.

Technique	Description	Critical issues
Z-Swab	Movement in a “Z” pattern applied to viable tissue with moderate pressure. Covers a wider area compared with the Levine technique.	High risk of contamination if not performed correctly; requires technical skill.
Levine	The swab is pressed onto a 1 cm^2^ area of viable tissue and rotated to collect wound fluid.	Potentially superficial sampling; limited sampling area.

##### 3.1.1.4 Standard operating procedure (SOP)

- Collect detailed clinical information about the patient (demographic data, medical history, comorbidities, ongoing therapies, allergies, hygienic conditions, etc.).
- Inform the patient and/or caregiver about the procedure (purpose, method, and potential risks).
- Use sterile, single-use devices (gauze, drape, gloves, swab).
- Irrigate the perilesional and lesional skin with sterile saline solution (avoid disinfectants, as they may compromise microbiological results, e.g., false-negative findings).
- Perform mechanical scraping to remove necrotic debris and slough.
- Cleanse the wound with sterile saline solution.
- Apply the selected sampling technique (Z-swab or Levine).
- Swab only well-cleaned viable tissue (exclude pus, necrosis, or fibrin).
- Verify and correctly label the specimen.
- Place the swab in a sterile container if not pre-packaged.
- Store at 4 °C for a maximum of 24 h if the sample is not immediately sent to the microbiology laboratory.
- Dress and close the wound according to standard procedures.

#### 3.1.2 Tissue biopsy

##### 3.1.2.1 General considerations

According to current international guidelines and expert recommendations, tissue biopsy is considered the gold standard for the microbiological diagnosis of deep infections in both chronic and acute wounds (16–18). It consists of collecting a tissue fragment approximately 3–4 mm in size from viable tissue at the wound bed, using sharp instruments (e.g., punch biopsy, scalpel, curette). This approach allows accurate quantitative and qualitative microbiological analysis, enabling identification of pathogens responsible for clinically significant infections and/or delayed wound healing (≥10^5^ CFU/g of tissue = infection) (16, 17). Traditionally, a bacterial load of ≥10^5^ CFU/g of tissue has been considered suggestive of infection. However, this threshold is not universal and should always be interpreted in conjunction with the clinical presentation, host factors, and microbiological findings.

##### 3.1.2.2 Indications for tissue biopsy

When should a tissue biopsy be performed?

Main indications include:

- Presence of deep or chronic infection (severe pain, extensive oedema, marked local warmth, presence of deep necrosis, abundant purulent exudate, malodour, signs of systemic infection);
- Failure of empirical therapy;
- Immunocompromised patients.

##### 3.1.2.3 Standard operating procedure (SOP)

(Table 2)

**Table 2:** Standard operating procedure (SOP) for tissue biopsy sampling.

Phase	Description
Data collection	Complete medical history (comorbidities drugs, allergies, hygienic conditions).
Patient Information and informed consensus	Clear communication with the patient or caregiver and acquisition of written informed consent (invasive procedure).
Preparation of sterile materials	Sterile gloves, forceps, biopsy punch, sterile containers, and gauze.
Wound cleansing	Cleansing of the wound with sterile normal saline solution (no antiseptics, as they may interfere with diagnostic results).
Removal of debris and necrotic tissue	Mechanical debridement (scraping) of the wound surface using sterile gauze moistened with sterile normal saline. Removal of fibrin, slough, or eschar.
Irrigation	Copious irrigation of the wound with sterile normal saline under moderate pressure.
Anaesthesia	Application of topical anaesthetics after assessment of potential allergies.
Specimen collection	Use of a biopsy punch, scalpel, or curette to obtain viable tissue from the wound bed.
Specimen preservation	Placement of the specimen in sterile normal saline or appropriate culture medium (formalin must not be used), with storage at 4 °C.
Transport to the laboratory	Immediate delivery or within 2 h; refrigerated samples may be stored for up to 24 h.
Post-procedural care	Local haemostasis, application of a clean dressing, and local monitoring.

#### 3.1.3 Fine needle aspiration

##### 3.1.3.1 General considerations

Fine needle aspiration (FNA) is a minimally invasive diagnostic–therapeutic procedure that involves the aspiration of purulent material from subcutaneous tissue or deep pockets using a sterile needle and a syringe containing sterile saline solution. It is occasionally used for the partial drainage of abscesses. Although FNA does not allow quantitative assessment of bacterial load, it is effective in identifying deep-seated microbial populations, including obligate anaerobes and enterobacteria (16–18). Owing to its minimally invasive nature, the procedure is generally well tolerated and more acceptable to patients.

##### 3.1.3.2 Indications for FNA

When should fine needle aspiration be performed?

Main indications include:

- Suspected deep or atypical infections;
- Diagnosis of localised infection (e.g., abscess, phlegmon);
- Partial drainage of an abscess for therapeutic purposes.

##### 3.1.3.3 Standard operating procedure (SOP)

Unlike swab or tissue biopsy, fine-needle aspiration requires the needle to pass through intact periwound skin before reaching the target tissue. Therefore, antisepsis of the intact skin is performed to reduce contamination of the aspirate by resident skin flora, whereas antiseptics are avoided on the wound surface before swab or tissue biopsy because they may interfere with microbiological recovery.

- Obtain a detailed patient history (demographic data, current medications, comorbidities, allergies, hygienic conditions);
- Inform the patient and/or caregiver about the procedure and obtain informed consent (minimally invasive procedure);
- Use sterile equipment (gloves, sterile drape, syringe, saline solution, gauze);
- Clean the skin surrounding the lesion with sterile saline solution;
- Disinfect the periwound skin—not the open wound—using 2% chlorhexidine or povidone–iodine;
- Aspirate 0.5 mL of sterile saline solution into the syringe;
- Insert the needle through intact skin towards the inflamed margin of the ulcer;
- Inject the saline solution and immediately re-aspirate it;
- Transfer the specimen into a sterile container with appropriate transport medium;
- Deliver the sample to the laboratory within 2 h of collection; if this is not possible, store it under refrigerated conditions for 24–48 h;
- If bleeding or exudate leakage occurs, perform gentle cleansing and apply a sterile dressing.

#### 3.1.4 Final considerations

Tissue biopsy is an invasive procedure and may cause pain and bleeding, particularly in patients receiving anticoagulant therapy; therefore, it is generally less well tolerated than swab sampling or fine needle aspiration. However, the diagnostic accuracy of tissue biopsy and fine needle aspiration (FNA) is higher than that of swab sampling, due to greater sample representativeness and reduced contamination by superficial colonising microorganisms (16–18).

Tissue biopsy and fine needle aspiration can only be performed by medical staff, with possible nursing support, whereas swab sampling can also be carried out by nursing personnel. It should also be noted that swab cultures may yield positive results due solely to contaminating organisms.

Overall, all three sampling techniques play a role in microbiological diagnostics when there is clinical suspicion of infection, and their use should be guided by the clinical context and diagnostic objectives (17–19).

**Microbiological processing of tissue biopsy specimens, wound swabs, exudates, and related samples**

Accurate microbiological diagnosis of skin, soft-tissue, and deep-seated infections depends on appropriate specimen selection, prompt transport, and timely laboratory processing. Rapid processing is important not only for preserving microbial viability but also for limiting post-collection changes in microbial composition, including overgrowth of rapidly replicating organisms, loss of fastidious species, and alterations in microbial burden that may affect the distinction between colonisation and infection (20, 21).

To facilitate the practical implementation of these principles, the SOPs presented in this document were compiled by integrating procedural recommendations from the available scientific literature, international guidance, and routine microbiological practice. Table 3 complements these procedures by summarizing the recommended transport, storage, and processing conditions for the different sampling methods and by emphasising the importance of prompt specimen transport and processing to minimise pre-analytical changes that may alter microbial viability, microbial burden and specimen composition, thereby preserving the reliability and clinical interpretation of microbiological findings.

**Table 3:** Summary of transport and storage conditions.

Sampling method	Transport	Storage (if delay is unavoidable)	Recommended processing time
Tissue biopsy	Sterile container, immediate transport	4 °C	as soon as possible, preferably within 2 h after collection
Fine-needle aspiration	Sterile container, immediate transport	4 °C	Process as soon as possible, preferably within 2 h after collection
Wound swab	Appropriate bacterial transport system (e.g., Amies medium); prompt transport	According to the transport system manufacturer's instructions until processing	Process as soon as possible, preferably within 2 h after collection

No evidence-based maximum processing time has been established. Immediate transport and processing are recommended. Refrigeration should only be considered when immediate processing is not feasible and should not be regarded as equivalent to immediate processing.

These recommendations highlight that the quality of microbiological diagnostics depends not only on the sampling technique but also on the appropriate management of specimens throughout the pre-analytical phase.

Whenever feasible, deep tissue specimens obtained by biopsy or aspiration are preferred over superficial swabs because they provide a higher diagnostic yield and correlate more closely with clinically significant infection, particularly in chronic wounds, diabetic foot infections, and surgical-site infections (22–24). Adequate tissue volume is essential because microorganisms are often unevenly distributed within infected tissues. Punch biopsy specimens of approximately 3–4 mm are commonly used and should be processed according to established microbiological procedures (25–29).

Tissue homogenisation represents a key pre-analytical step in the processing of biopsy specimens. By disrupting the tissue matrix and generating a uniform homogenate, homogenisation enhances microbial recovery from infected tissue and facilitates the detection of organisms that may otherwise remain embedded within the specimen, including fastidious and anaerobic microorganisms (22, 30–33). Homogenisation also enables quantitative culture, allowing microbial burden to be expressed as colony-forming units per gram of tissue (CFU/g), which may assist in the assessment of infection severity and the interpretation of microbiological findings (24, 33).

Following homogenisation, specimens are typically examined by direct microscopy and cultured using standard microbiological methods. Gram staining can provide rapid information regarding bacterial morphology and the presence of inflammatory cells, whereas quantitative culture techniques may be applied when assessment of microbial burden is clinically relevant. Identification of recovered microorganisms is commonly performed using automated platforms and/or matrix-assisted laser desorption ionisation time-of-flight mass spectrometry (MALDI-TOF MS). Antimicrobial susceptibility testing should be undertaken according to current EUCAST or CLSI recommendations, and molecular detection of resistance determinants, including mecA, vanA/vanB, and carbapenemase genes, may provide complementary information when interpreted alongside conventional culture results (34–37).

Recent advances in molecular diagnostics, including 16S rRNA gene sequencing, shotgun metagenomics, and microbiome profiling, are expanding the understanding of chronic wound microbiology and may complement conventional culture by improving the detection of polymicrobial communities and fastidious microorganisms, although further standardisation is required before widespread implementation in routine clinical practice (38).

For wound swabs, pus specimens, exudates, and irrigation fluids, Gram staining remains a useful adjunctive investigation when available. Culture should include both aerobic and anaerobic conditions and employ selective and non-selective media according to the clinical context. In selected situations, particularly when specimen volume is limited or fastidious organisms are suspected, inoculation into blood-culture bottles may improve microbial recovery and increase diagnostic yield (39).

Culture protocols should employ microbiological media and incubation conditions appropriate to the suspected pathogens and clinical setting. Recovery of aerobic, anaerobic, fungal, and fastidious microorganisms may require different atmospheric conditions and, in selected cases, prolonged incubation (32, 40)_._

Microbiological findings should always be interpreted within the clinical context, taking into account specimen quality, sampling method, host factors, and the distinction between contamination, colonisation, and true infection. Close communication between clinicians and microbiology laboratories is essential to optimise diagnostic accuracy, support antimicrobial stewardship, and guide patient management (20, 22, 24).

### 3.2 Interpretation of microbiological findings in chronic wounds

#### 3.2.1 Distinguishing colonisation from infection

Microbiological data obtained from chronic wound samples have no absolute value and must always be interpreted within the clinical and morpho-functional context of the lesion. Ulcer surfaces are inevitably colonised by microbial communities, including commensal organisms, opportunistic species and potential pathogens. For this reason, the diagnosis of infection is primarily clinical, while microbiological analysis serves to confirm the aetiology and guide targeted antimicrobial therapy.

Colonisation is defined as the presence of microorganisms in the absence of clinical or laboratory evidence of infection. This condition is common in chronic wounds and does not require antimicrobial treatment; rather, it should be managed through optimal local wound care (such as cleansing, debridement, moisture balance and the use of advanced dressings) together with close clinical monitoring.

Infection is characterised by the presence of local clinical signs (e.g., erythema, oedema, warmth, pain, malodour, purulent exudate or necrosis) and/or systemic signs (fever, leucocytosis, elevated inflammatory markers), in association with microbiological identification of pathogens in clinically significant concentrations or with concordant instrumental findings.

Current guidelines recommend, whenever feasible, the collection of deep tissue samples (biopsy or curettage) to improve diagnostic accuracy and enhance the therapeutic relevance of microbiological results (40–43).

#### 3.2.2 Quantitative Thresholds and their clinical interpretation

The traditional cut-off of ≥10^5^ CFU/g of viable tissue, as previously described, derived from historical quantitative bacteriology, is associated with an increased risk of infection and tissue damage; however, it is not universal and must always be integrated with the clinical presentation, anatomical site, host status and the biology of biofilm. Certain pathogens—such as Staphylococcus aureus, Pseudomonas aeruginosa and group A β-haemolytic streptococci—may cause clinically significant infection even at lower bacterial loads, particularly in critical anatomical sites or in immunocompromised patients. Sampling technique (depth, prior debridement and timing) and the presence of biofilm substantially influence quantitative results and their interpretation.

Confocal laser scanning microscopy and fluorescence *in situ* hybridisation (FISH) have demonstrated multispecies biofilms in a substantial proportion of chronic ulcers. This finding helps explain the discrepancy between *in vitro* antimicrobial susceptibility (based on planktonic cells) and clinical response, and supports the concept that the distinction between colonisation and infection is eco-systemic, dependent on the functional role of microorganisms and their interaction with the host. Rational antimicrobial use therefore requires an integrated clinical–microbiological approach, consistent with international stewardship frameworks.

#### 3.2.3 Clinical Contextualisation of the antibiogram in chronic wounds

The antibiogram is an essential tool for guiding antimicrobial therapy; however, its clinical interpretation requires a critical appraisal that goes beyond the standard susceptible/intermediate/resistant (S/I/R) classification. In chronic wounds in particular, the gap between *in vitro* susceptibility data and *in vivo* therapeutic effectiveness may be substantial, owing to the eco-biological complexity of the wound microenvironment.

#### 3.2.4 Tissue Penetration and local perfusion

Antibiotic distribution within tissues is influenced by anatomical and pathophysiological factors. In ischaemic chronic wounds or in conditions associated with impaired microcirculation (such as diabetic foot ulcers or chronic venous leg ulcers) tissue concentrations may be significantly lower than plasma levels, even when the minimum inhibitory concentration (MIC) appears favourable. It is therefore essential to select agents with good bioavailability and effective diffusion into peripheral tissues (e.g., fluoroquinolones, linezolid, clindamycin) and to consider adjunctive strategies, such as surgical revascularisation or hyperbaric oxygen therapy, to enhance tissue penetration.

#### 3.2.5 Presence of biofilm

Biofilm formation is a major contributor to impaired healing in chronic ulcers. The extracellular matrix of biofilm creates gradients of oxygen, pH and nutrients that confer antibiotic tolerance up to 100–1,000 times greater than that observed in planktonic cells. Certain antibiotics (e.g., rifampicin, fluoroquinolones, tetracyclines) penetrate the biofilm matrix more effectively; nevertheless, biofilm eradication almost invariably requires combined strategies, including mechanical debridement, targeted antimicrobial therapy and the use of dressings with anti-biofilm activity (such as silver, iodine or advanced antiseptics). Standard antibiograms, which are based on planktonic cultures, do not adequately reflect true antimicrobial susceptibility in the presence of biofilm.

#### 3.2.6 Pharmacokinetic/pharmacodynamic (PK/PD) temporal dynamics

Therapeutic success depends on the interaction between pharmacokinetic properties (drug concentration achieved at the site of infection) and pharmacodynamic effects (bacteriostatic or bactericidal activity). Different antibiotic classes are associated with distinct PK/PD targets. Consequently, the simple categorisation of an isolate as “susceptible” may be insufficient, as an antibiotic deemed susceptible may fail to achieve adequate exposure at the infection site if appropriate dosing regimens are not applied.

#### 3.2.7 Polymicrobial Infection and microbial interactions

Chronic wound infections are frequently polymicrobial, involving aerobic and anaerobic bacteria as well as yeasts. Microbial interactions may be synergistic (e.g., *S. aureus* and *P. aeruginosa* within co-existing biofilms) or antagonistic, thereby modifying the therapeutic response. As the antibiogram evaluates individual isolates in isolation, it may under- or overestimate clinical efficacy. From a clinical perspective, initial empirical therapy should cover the dominant pathogens, while subsequent de-escalation must be tailored to the actual clinical and microbiological dynamics.

#### 3.2.8 Acquired resistance profiles

The increasing prevalence of multidrug-resistant organisms (e.g., MRSA, ESBL-producing Enterobacterales, VRE, carbapenemase-producing bacteria) in chronic wounds represents a growing challenge. Antibiogram interpretation should integrate known resistance mechanisms with local epidemiological data (laboratory surveillance, ECDC reports) and, where available, rapid molecular methods (e.g., PCR detection of resistance genes, shotgun metagenomics). This integrated approach enables earlier optimisation of antimicrobial therapy, reduces unnecessary broad-spectrum antibiotic use and helps limit selective pressure (18, 44).

## 4 Discussion

The present recommendations address a critical gap in the clinical management of hard-to-heal wounds, namely the lack of standardisation in microbiological sampling and interpretation. Despite the availability of international recommendations, including those from the European Wound Management Association, the International Wound Infection Institute, and the Infectious Diseases Society of America, real-world clinical practice remains highly heterogeneous, particularly with regard to sampling techniques, timing of collection, and integration of microbiological findings into clinical decision-making. This variability contributes to diagnostic uncertainty, inappropriate antimicrobial use, and suboptimal patient outcomes.

A central contribution of this work is the development of a structured and operational framework aimed at reducing such variability through the standardisation of microbiological sampling procedures. Unlike existing guidance, which often provides general recommendations, this document translates available evidence into detailed SOPs, thereby facilitating implementation across different healthcare settings, including hospital, outpatient, and community care. This operational focus represents a key strength of the document and responds to a well-recognised need for reproducibility and procedural clarity in wound care practice.

The selection of sampling technique emerges as a critical determinant of diagnostic accuracy. The comparative analysis of swab sampling, tissue biopsy, and fine needle aspiration confirms that each method has distinct advantages and limitations. Tissue biopsy remains the reference standard for the diagnosis of deep infections due to its higher specificity and ability to provide quantitative microbiological data (16–18). Fine needle aspiration offers a minimally invasive alternative for the investigation of deep-seated or localised infections, while swab sampling—despite its lower specificity and susceptibility to contamination—retains an important role due to its feasibility and accessibility in routine practice (17–19). These findings support a context-driven approach, in which the choice of technique is guided by clinical suspicion, anatomical depth of infection, and patient-related factors, rather than a rigid hierarchical model.

The interpretation of microbiological findings represents another major challenge in the management of chronic wounds. The distinction between colonisation and infection is inherently complex, as chronic wounds are invariably colonised by diverse microbial communities. As previously conceptualized (43, 44), the mere presence of microorganisms does not equate to infection, and microbiological data must always be interpreted within the broader clinical and host context. This document reinforces the principle that infection remains primarily a clinical diagnosis, with microbiological analysis serving a confirmatory and therapeutic guidance role. The traditional quantitative threshold of ≥10^5^ CFU/g of tissue, although historically useful, is insufficient when applied in isolation, particularly in the presence of virulent pathogens or in immunocompromised patients.

An important aspect highlighted in this work is the role of microbial ecology and biofilm in shaping infection dynamics. Biofilm contributes to antimicrobial tolerance, persistence of infection, and discrepancies between *in vitro* susceptibility testing and clinical response (8–10). These findings support integrated management strategies combining appropriate microbiological diagnosis, debridement, targeted antimicrobial therapy, and optimal wound care interventions.

The limitations of standard microbiological techniques are further compounded by the challenges associated with antibiogram interpretation in chronic wound settings. As highlighted in previous studies (18, 44), the conventional susceptible/intermediate/resistant (S/I/R) classification does not fully capture the complexity of antimicrobial effectiveness *in vivo*. Factors such as tissue penetration, local perfusion, pharmacokinetic/pharmacodynamic (PK/PD) relationships, and the presence of biofilm significantly influence therapeutic outcomes. In particular, impaired microcirculation in conditions such as diabetic foot ulcers or chronic venous insufficiency may prevent adequate antibiotic concentrations from being achieved at the site of infection, even when laboratory susceptibility is favourable. This reinforces the need for a clinically contextualised interpretation of microbiological data, integrating laboratory findings with patient-specific and pathophysiological factors.

Polymicrobial infection represents an additional layer of complexity. Chronic wounds frequently harbour mixed microbial communities, including aerobic and anaerobic bacteria, as well as fungal species. Interactions between microorganisms may be synergistic or antagonistic, influencing both pathogenicity and response to treatment. As standard microbiological analysis evaluates individual isolates in isolation, it may fail to reflect these dynamic interactions, potentially leading to underestimation or overestimation of clinical relevance. From a therapeutic perspective, this supports an initial empirical approach targeting the most likely pathogens, followed by de-escalation based on clinical evolution and microbiological results.

The increasing prevalence of antimicrobial-resistant pathogens further complicates the management of chronic wound infections (18, 44). In this context, accurate and representative microbiological sampling is essential not only for guiding individual patient management but also for informing local epidemiological surveillance and antimicrobial stewardship strategies. The integration of conventional microbiology with advanced diagnostic tools, including molecular methods, may further enhance the timely detection of resistance mechanisms and support more targeted therapeutic interventions.

Overall, this document supports an integrated clinical–microbiological approach to chronic wound management, in which standardized sampling procedures, appropriate interpretation of microbiological findings, and antimicrobial stewardship are key components. By providing practical recommendations and standardized operating procedures, this document aims to improve diagnostic consistency and facilitate the implementation of evidence-informed microbiological practices across different healthcare settings.

## 5 Medico-legal implications

### 5.1 Medico-legal context

Healthcare-associated infections have long represented an important source of medico-legal litigation worldwide (45–55). Over recent decades, increasing attention to patient safety, antimicrobial resistance, and healthcare quality has reinforced the need for standardized diagnostic procedures and accurate documentation. In this context, standardized microbiological sampling is not only essential for appropriate clinical management but also plays an important role in specimen traceability, quality assurance, and medico-legal accountability.

### 5.2 Medico-legal litigation with high rates of adverse rulings against healthcare providers

Healthcare-associated infections (HAIs) represent the most frequent causes of medico-legal litigation and are associated with the highest rates of adverse judgments against healthcare systems. A study by Marrone et al. (56) analyzed compensation claims for alleged medical negligence related to HAIs in Italy. The findings showed that 90.2% of cases involved surgical site infections (SSIs), with coagulase-negative staphylococci (34.1%) and *Staphylococcus aureus* (24.4%) being the most frequently implicated pathogens. Failure to implement, or non-adherence to, HAI prophylaxis and/or prevention protocols emerged as the most common reason for acceptance of compensation claims. A limitation of the study is that the analyzed court rulings were selected randomly.

Surgical site infections are a major focus of surveillance by the European Centre for Disease Prevention and Control (ECDC), which publishes periodic reports on this topic. In the most recent report covering the period 2018–2020 (57) *Staphylococcus aureus* was identified in 15.2% of cases and coagulase-negative staphylococci in 14%. However, at the European level, the most frequently isolated pathogens were *Enterococcus* spp. (17.6%) and *Escherichia coli* (17.2%).

When these epidemiological data are compared with medico-legal evidence, they suggest that infections caused by pathogens more readily attributable to operator-related contamination—particularly staphylococci—have an almost twofold higher likelihood of leading to litigation and of resulting in successful claims.

#### 5.2.1 Medico-legal framework and evidentiary burden

Although the following discussion refers to selected Italian legal rulings, these are presented as illustrative examples of how national legal frameworks may reinforce the importance of standardized microbiological sampling. The underlying principles of appropriate documentation, specimen traceability, quality assurance, and adherence to standardized procedures are broadly applicable across different healthcare systems and jurisdictions.

The judgment of the Italian Supreme Court No. 6386/2023 (58) —widely known among professionals as the “*Travaglino ruling*”—has further exacerbated the well-described by Marrone et al. (56) legal exposure of healthcare institutions (and, consequently, of healthcare professionals), by imposing 13 specific evidentiary obligations to demonstrate that infection prevention measures have been duly implemented.

The issue is that such procedures are scarcely modifiable by the healthcare worker, and while the medical and nursing directors do possess such authority, it applies to only 3 of the 13 obligations.

Accordingly, it is no longer sufficient to have written guidelines on hygiene, sanitation and cleaning—even when supported by emergency procedures. Preventive actions must be certified with date and time of execution.

Furthermore, such guidelines, best practices and protocols must be formally disseminated and made known to all staff, as this dissemination itself falls within individual (subjective) obligations, as follows:

- The senior executive is required to define precautionary rules and bears the power–duty of supervision and verification (e.g., periodic meetings and inspections), similarly to the Hospital Infection Control Committee;
- The Medical Director is responsible for their implementation, for organising hygienic and technical–sanitary measures, and for supervising compliance with the provided instructions;
- The unit director (head of department, head of operational unit, ward manager), is the final executor of protocols and guidelines and is liable for failure to acquire accurate information on initiatives undertaken by other physicians or for failure to report deficiencies to the responsible authorities, even in relation to potential risks.

In addition, a more recent ruling of the Civil Supreme Court (59) on this matter has reaffirmed that “the burden of proof of all constitutive elements of the liability of the healthcare institution lies with the injured party”, while also stating that such proof “may be provided […] through simple presumptions”. Consequently, liability may no longer be established solely on objective evidence, but also on inferential assumptions, whose probative value will then be assessed by the court-appointed expert (CTU).

For these reasons, the early and correct execution of microbiological swab sampling represents a key safeguard for healthcare providers managing patients who may have acquired an infection in another facility.

#### 5.2.2 Risk of false-negative or false-positive results: technique- and operator-dependent factors

The risk of error in diagnostic testing cannot be determined *a priori*; however, it can be significantly reduced through two key measures:

- Formal adoption of these recommendations as institutional guidelines at ward, hospital or organisational level.
- Implementation of certified training programmes to validate competency in performing sampling procedures—at a minimum, wound swab collection—organised by recognised professional associations.

A certified operator who adheres to validated guidelines is demonstrably more competent than an *ad hoc* practitioner, and their actions carry greater clinical and medico-legal value.

Consequently, the entire procedure requires certification and standardization at every stage to maintain rigorous accuracy, explicitly detailing:

- Clear documentation of the sampling process, specifying the methodology used.
- Full traceability of the specimen and of every phase of the process, including transport dates and times.
- Assurance that the specimen is properly preserved and remains accessible for any subsequent analyses.

Only in this way will the medico-legal validity of the test be firmly established and difficult to challenge.

#### 5.2.3 Key medico-legal considerations

From a medico-legal perspective, standardized microbiological sampling should always be accompanied by accurate documentation of the procedure, including the indication for sampling, sampling technique, date and time of collection, anatomical site, transport conditions, and laboratory processing. Such documentation enhances specimen traceability, supports quality assurance, facilitates the interpretation of microbiological findings, and provides objective evidence that appropriate diagnostic procedures have been followed in accordance with antimicrobial stewardship principles.

## 6 Future directions

### 6.1 Artificial intelligence in microbiological diagnostics and infection assessment of hard-to-heal wounds

Artificial intelligence (AI), encompassing machine learning (ML), deep learning (DL), and natural language processing (NLP), is increasingly being explored as a tool to support infection diagnostics in chronic wounds, complementing rather than replacing conventional microbiological sampling. Distinguishing colonisation from clinically significant infection remains a central diagnostic challenge in wound care, and AI-based approaches are being investigated as adjuncts to clinical judgement in this area. Emerging applications include the analysis of biofilm architecture, microbiological data, and antimicrobial resistance patterns, with the potential to support diagnostic decision-making and antimicrobial stewardship.

Biofilm represents a major determinant of diagnostic and therapeutic failure in chronic wounds, and its detection by conventional culture remains limited. AI-assisted quantification tools, such as SEMTWIST, combine scanning electron microscopy with machine learning and demonstrate strong correlation with gold-standard peptide nucleic acid fluorescence *in situ* hybridisation (PNA-FISH) assessment (60). Such approaches may help bridge the persistent gap between standard culture-based diagnostics, which are typically based on planktonic organisms, and the true burden of biofilm-associated infection, which is not adequately captured by conventional antibiogram interpretation.

Recent systematic reviews have highlighted the potential of AI-based image analysis for the detection and classification of wound infections, including surgical site infections and diabetic foot ulcers. For example, deep learning models have demonstrated diagnostic accuracy comparable to that of clinicians in recognising infection-related features in wound images, although external validation and standardisation remain important barriers to clinical implementation (61, 62). These tools may support early recognition of infection, particularly in community or telemedicine settings, where access to specialist wound care is limited. However, their current role remains adjunctive, and they should not replace standardised microbiological sampling when infection is clinically suspected.

AI applied to metagenomic sequencing enables high-resolution, culture-independent profiling of wound microbiota, with the potential to identify polymicrobial communities, fastidious or unculturable organisms, and antimicrobial resistance determinants that may not be detected by standard culture-based methods (63). This is particularly relevant in the interpretation of polymicrobial infections and in refining empirical antimicrobial choices before culture results become available. In parallel, NLP applied to electronic health records may support automated extraction of infection-related clinical and microbiological information, facilitating structured documentation, epidemiological surveillance, and antimicrobial stewardship programmes at institutional level.

From a regulatory and medico-legal standpoint, AI-based diagnostic tools intended for clinical use in the European Union fall under the Medical Device Regulation (MDR 2017/745) and the AI Act (EU Regulation 2024/1689), requiring CE marking and adherence to harmonised standards. Clinicians retain final responsibility for the interpretation of microbiological and AI-derived findings, and the use of non-validated tools should not substitute for standardised microbiological sampling and laboratory diagnostic pathways.

Despite this promise, clinical translation of AI in infection diagnostics remains constrained by important limitations, including reliance on retrospective, single-centre datasets, limited external validation, and heterogeneity in image acquisition or sequencing protocols across studies. Prospective, multicentre validation against standard microbiological reference methods is needed before these tools can be recommended for routine integration into diagnostic pathways. Nonetheless, AI-assisted infection recognition, biofilm quantification, and metagenomic profiling represent a promising direction for complementing standardised microbiological sampling and supporting antimicrobial stewardship in the management of hard-to-heal wounds.

### 6.2 Integrating a one health approach into the management of hard-to-heal wounds: operational implications for clinical practice

The management of hard-to-heal wounds requires an integrated approach that extends beyond individual patient care, encompassing the complex interaction between host factors, microbial communities, healthcare practices, and environmental determinants. Within this framework, the One Health perspective can be translated into clinical practice through the alignment of microbiological sampling, antimicrobial stewardship, and epidemiological surveillance systems.

Microbiological sampling in chronic wounds should not be regarded solely as a diagnostic procedure, but also as a structured source of epidemiological data. Variability in sampling techniques, pre-analytical handling, and laboratory processing has been identified as a major limitation affecting the reliability and comparability of microbiological findings across different healthcare settings (10–12). The standardisation of sampling procedures, including the choice of technique, timing of collection in relation to debridement, and transport conditions, represents a critical step in generating consistent and reproducible data. When systematically documented and integrated into laboratory workflows, these data can contribute to local surveillance systems, enabling the monitoring of pathogen distribution and antimicrobial resistance patterns in line with antimicrobial stewardship priorities (18, 44).

A key operational component of the One Health framework is the direct integration of microbiological sampling with antimicrobial stewardship strategies. Appropriate microbiological investigations should support rational antimicrobial prescribing, including the initiation, adjustment, or discontinuation of antimicrobial therapy, and should always be interpreted in conjunction with the clinical assessment. This approach also requires consideration of local epidemiological data, particularly in settings with a high prevalence of multidrug-resistant organisms such as methicillin-resistant *Staphylococcus aureus* or extended-spectrum β-lactamase-producing *Enterobacterales* (18, 44).

The environmental and organisational context in which chronic wound care is delivered represents an additional determinant of infection risk. Patients are often managed across a continuum that includes hospital wards, outpatient clinics, long-term care facilities, and home environments, each characterised by specific hygiene standards, care practices, and potential sources of contamination. The operational implementation of a One Health approach therefore requires strict adherence to infection prevention and control measures, including appropriate hand hygiene, aseptic dressing techniques, and adequate sterilisation of equipment. At the same time, continuous training of healthcare personnel in both wound care and microbiological sampling procedures is essential to ensure consistency and reduce variability in clinical practice. These measures play a crucial role in limiting pathogen transmission and reducing the burden of healthcare-associated infections.

From a biological perspective, chronic wounds should be understood as dynamic microbial ecosystems in which interactions between host tissues and microbial communities influence healing trajectories (6, 7). The presence of biofilm in chronic wounds further complicates infection dynamics by promoting microbial persistence and antimicrobial tolerance (8–10). This ecological understanding has direct clinical implications. Antimicrobial treatment should not be initiated in the absence of clear clinical signs of infection, while greater emphasis should be placed on optimising local wound care strategies, including debridement, moisture balance, and the use of advanced dressings capable of modulating the wound microenvironment. Recognising the role of biofilm and polymicrobial interactions is essential for explaining treatment failure and for guiding more effective, integrated therapeutic approaches. In this context, infection should be interpreted not simply as the presence of microorganisms, but as the result of a complex interaction between microbial activity and host response (43).

The effective implementation of a One Health approach in chronic wound management ultimately depends on the integration of clinical, microbiological, and epidemiological data into coordinated systems of care. Standardised microbiological sampling provides the foundation for generating high-quality data that can be aggregated across institutions and healthcare settings. Such integration enables more accurate surveillance of antimicrobial resistance trends and supports more informed clinical decision-making. Advances in diagnostic technologies, including molecular methods, may further enhance the detection of resistance mechanisms and improve the characterisation of complex microbial communities (18, 44). By linking standardised sampling procedures with surveillance and stewardship strategies, the One Health framework can be operationalised as a practical tool that improves both individual patient management and broader public health outcomes.

## 7 Conclusions

Chronic and hard-to-heal wounds represent a growing clinical and public health challenge, in which accurate infection diagnosis remains a critical determinant of patient outcomes. The present document provides a structured and operational framework for microbiological sampling, addressing a major gap in the standardisation of clinical practice. By integrating evidence-based recommendations with detailed standard operating procedures, this document facilitates a more consistent, reproducible, and clinically meaningful approach to wound microbiology across diverse healthcare settings.

A key message emerging from this work is that microbiological findings must not be interpreted in isolation, but rather within an integrated clinical–microbiological model that accounts for host factors, microbial ecology, and the complex dynamics of biofilm and antimicrobial resistance. Standardisation of sampling techniques, combined with critical interpretation of laboratory data, is essential to improve diagnostic accuracy and support appropriate antimicrobial use.

Beyond its immediate clinical implications, this framework aligns with broader priorities in antimicrobial stewardship, infection control, and health system resilience. The integration of emerging technologies and interdisciplinary approaches further highlights the need for adaptive and system-oriented models of care. Collectively, these recommendations aim to bridge the gap between theoretical guidance and real-world practice, ultimately contributing to improved patient outcomes and more sustainable management of chronic wound infections.
